# Using the Optical Mouse Sensor as a Two-Euro Counterfeit Coin Detector

**DOI:** 10.3390/s90907083

**Published:** 2009-09-04

**Authors:** Marcel Tresanchez, Tomàs Pallejà, Mercè Teixidó, Jordi Palacín

**Affiliations:** Department of Computer Science and Industrial Engineering, University of Lleida, Jaume II, 69, 25001 Lleida, Spain; E-Mails: mtresanchez@diei.udl.cat (M.T.); tpalleja@diei.udl.cat (T.P.); mteixido@diei.udl.cat (M.T.)

**Keywords:** optical sensor, optical mouse sensor, vision based counterfeit

## Abstract

In this paper, the sensor of an optical mouse is presented as a counterfeit coin detector applied to the two-Euro case. The detection process is based on the short distance image acquisition capabilities of the optical mouse sensor where partial images of the coin under analysis are compared with some partial reference coin images for matching. Results show that, using only the vision sense, the counterfeit acceptance and rejection rates are very similar to those of a trained user and better than those of an untrained user.

## 1. Introduction

A counterfeit coin is an imitation of a genuine coin made with the intent to defraud. Currently, automatic counterfeit coin detection in cash or vending machines is based on the use of low cost sensors to measure some physical coin properties [1] such as weight, size, thickness, conductivity [2], magnetic [3] or acoustic properties [4,5]. Alternatively, when the physical properties of the coins (weight, size) are very similar, image based coin recognition can be used. For example, image based recognition was applied to ancient coin classification in [6], and in [7] coin image recognition using image abstraction and spiral decomposition was proposed to extract features such as scale, translation, and rotation invariant information. In [8–10] image based coin recognition was performed using template matching and neural networks. In [10] entire side coin images were acquired in a reduced set of degrees and two coins were classified using neural networks: the two-Euro and a similar coin from other country, with a 96.3% of success. In [11] an image based coin classificatory system able to discriminate between coins from 30 countries using eigenspaces was presented. The system uses two high resolution cameras to acquire complete image of both sides of the coins and get rotational invariance parameters as thickness and diameter with a correct classification rate of 93.23%.

In this work a new image-based approach is proposed to detect counterfeit coins. The approach is based on the image acquisition capabilities of the optical mouse sensor originally designed as a non-contact motion sensor to replace the mechanical wheels of the computer mouse. The optical mouse sensor includes a digital signal processor (DSP), a CMOS camera, lens, and a led or laser-led self illumination system. The internal DSP is used to apply some specific image processing algorithms for motion measurement and to control the illumination of the image acquired by the sensor in a closed loop. The optical mouse sensor is optimized to acquire high contrast roughness images of plain objects at a very short distance and has other applications than a human computer interface device. In [12] it was used as a two-dimensional displacement sensor and applied in [13] to mobile robot dead-reckoning, although showing some limitations when used as a displacement sensor [14,15]. Recently, in [16] the optical sensor was used as an image acquisition device and in [17] the acquisition capabilities of optical sensor were used to build an incremental rotary encoder with up to 1,900 pulses per turn.

In this work the optical sensor is used as an image acquisition device in combination with a dedicated microprocessor allowing the development of very compact vision based measurement systems. The main advantage of this new approach is the low cost of the optical sensor but the main disadvantage is that the image acquired covers a very small frontal area (1/14 of a two-Euro coin).

This work is focused on the two-Euro counterfeit coin detection because it is the more valuable and more falsified coin in the European Union (EU), accounting for 79% of the total counterfeits detected in 2008 [18]. The proposed counterfeit detector is planned as a complimentary detector rather than an isolated system. Specifically, the system must detect the coins from non EU countries that have similar size and weight to the two-Euro coin. They are not imitations or copies of the two-Euro coin but they are also used with the intent to defraud and thus are considered counterfeit coins in this work.

The paper is structured as follows. Section 2 shows the history of the counterfeit Euro coins. Section 3 describes the working principle of the optical mouse sensor. The complete system for counterfeit detection is described in Section 4. Section 5 shows the experimental validation of the counterfeit system. Finally, Section 6 presents the conclusions of this paper.

## 2. Background

The euro (labeled €) was launched as an invisible currency on 1 January 1999, when it became the currency of more than 300 million people in Europe. Euro cash was introduced in 1 January 2002, replacing the banknotes and coins of the national currencies like the Belgian franc, the Deutsche Mark and the Spanish Peseta [19,20].

**Figure 1.**
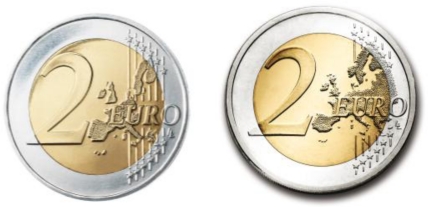
Common side of all two-Euro coins minted before 2007 (left) and minted from 2007 onwards (right).

The Euro coin series comprises eight different denominations: 1, 2, 5, 10, 20 and 50 cent, €1 and €2. They are all different in terms of size, weight, material, color and thickness to facilitate recognition by the blind and the partially sighted. The Euro coins have a common reverse side (designed by Mr. Luc Luycx of the Royal Belgian Mint) showing the value and a map of Europe, and a national side having 19 different designs and 48 commemorative national series with specific local representations [20]. The common sides of the coins were modified on 7 June 2005 by the Council to include the new Member States of the EU. Figure 1 shows the two common reverse sides available for the two-Euro coin.

The highest denomination euro coin, the two-Euro, is bi-metallic with two different metals fused together, showing two different colors (Figure 1) to hinder counterfeiting. The inner part (gold color) is made of three layers: nickel brass, brass and nickel brass. The outer silver colored part is made of cupronickel (Figure 1). They have a diameter of 25.75 mm, a 2.20 mm thickness and a mass of 8.5 grams. The edges of the coin also vary between national issues but most of them are finely ribbed with edge lettering.

**Figure 2.**
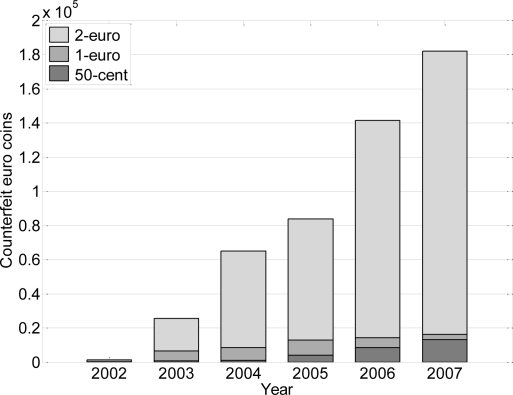
Counterfeit Euro coins detected in circulation up to 2007.

In July of 2008 the European Anti-Fraud Office (OLAF) [21] of the European Commission reported the number of counterfeit coins found up to 2007 (Figure 2). The total number of counterfeit euro coins removed from circulation reached 211,100, mainly in Germany. Figure 2 shows that the two-Euro coin is the most falsified, accounting for over 85% of the total counterfeits detected in 2007.

**Figure 3.**
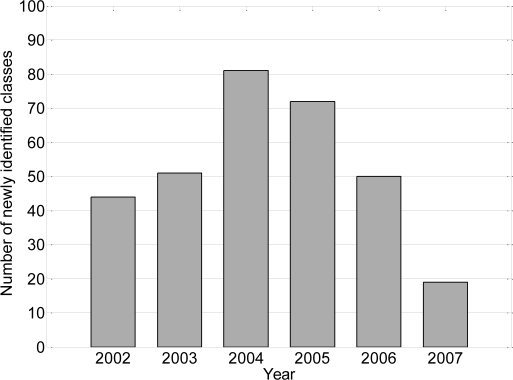
Number of newly two-Euro counterfeit common classes.

According the OLAF [21], counterfeit coins can be divided into two classes: common classes (stamped counterfeits) and local classes (cast counterfeits). Common classes are counterfeits made with a stamping process, similar to the one used in official minting, and can produce large amounts of counterfeits. Local classes are counterfeits of relatively low quality and quantity and should therefore be considered as less dangerous. The proportion of local classes in the total quantity of counterfeits registered is continuously decreasing; in 2007 it was 0.13%, which is very low. Figure 3 shows the number of newly two-Euro counterfeit common classes detected by the European Technical and Scientific Centre (ETSC) for each year since the introduction of euro coins.

Apart from the common and local classes, there are legal coins stamped in countries around the world with very similar shape, weight and size than the two-Euro coin, but with a fraction of its value. Currently, the coins from four nations (which are not listed intentionally) have appeared in vending machines throughout Europe. All these coins do not have the common side of the two-Euro coin and can be easily identified with a simple visual inspection. Therefore, a vision-based counterfeit detector must also reject all these coins based of the differences on the stamped images and relieves of the coins.

## 3. The Optical Mouse Sensor

**Figure 4.**
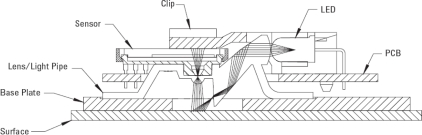
Sectional view of assembly components for the ADNS-3088 sensor (courtesy of Avago).

The optical mouse sensor is based on a very compact image acquisition system that consists of a high speed CMOS photodetector array [22], an infrared light source (LED) that illuminates the surface, and a convex lens that collects the reflected light (Figure 4). This device is placed very close to the surface (nominal distance of 2.4 mm) and detects small variations in the roughness of the surface by means of the shadows enhanced by the lateral infrared light. The optical mouse sensor compares images of the surface at a very high rate, over 6,400 frames per second, to returns relative X and Y axes motion.

The ADNS-3088 [23] optical mouse sensor from Avago Technologies was used in this paper; it includes a DSP and a CMOS camera of 30 × 30 pixels on the same chip. According to the manufacturer, the maximum measurable speed is 40 inches per second (ips) (1.016 m/s), the maximum acceleration during measurements is 15 G (147.15 m/s^2^) and the selectable resolutions are 400 and 800 counts per inch (cpi). The communication with the optical sensor is performed by the standard SPI bus using 8 bit of address and 8 bits of data. A low cost microprocessor, such as the PIC18F4550 working at 48 MHz, requires 130 μs to complete the access to one register.

The ADNS-3088 provides read and write access to 31 internal registers [23]. Apart from the common registers available for relative motion measurement: MOTION, DELTA_X, and DELTA_Y, the optical mouse sensor also has other interesting registers, such as PIXEL_BURST that allows sequential access (pixel by pixel with values from 0 to 63) to the image captured by the sensor (Figure 6), and SQUAL, SHUTTER, and PIXELSUM that are internal registers used to control the illumination of the area under the CMOS camera [17].

**Figure 5.**
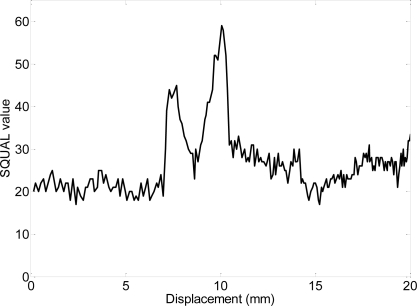
Dynamic evolution obtained from the SQUAL register when the optical mouse sensor is moving over a white paper with a transversal black line of 1.2 mm width.

**Figure 6.**
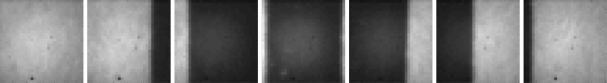
Some images captured by the ADNS-3088 optical mouse sensor when moving over a black line of 1.2 mm width.

Figure 5 shows an example of the dynamic evolution of the values of the SQUAL register obtained when moving the optical sensor over a black line painted on a white surface; when the black line appears in the image the SQUAL value increases suddenly; when the black line covers most part of the image the SQUAL value decreases to its average value; finally, when the black line is disappearing the SQUAL value increases again suddenly returning to the average value when the line has complete disappeared from the image. Figure 6 shows some images acquired from the optical sensor ADNS-3088 during this experiment.

## 4. Two-Euro Counterfeit Coin Detection

**Figure 7.**
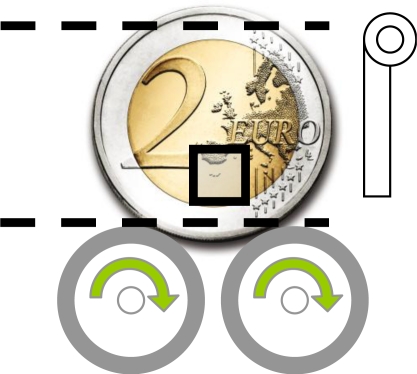
Schematic view of the proposed two-Euro counterfeit coin detector. The square is a representation of the area of the image acquired by one optical sensor.

Figure 7 shows a schematic view of the proposed two-Euro counterfeit coin detector, which contains two rotating wheels in contact with the coin under test; two guides (dotted lines) to fix the vertical orientation of the coin; a mechanical plug to eject the coin after the test; and one optical sensor in each side of the coin (the area of the image acquired is labeled with a rectangle for reference). The distance from the optical sensor to the side of the coin is the recommended by the manufacturer (2.4 mm).

The image acquired by the optical sensor has a very low resolution (30 × 30 pixels) but covers a very small area of the coin so the small details (relief information) are clearly revealed. The coin under test is rotated using the two additional rotating wheels to get a sequence of detailed images of the coin. The time spent in the complete rotation of the coin is the most time consuming operation but, in theory, it can be optimized very much because the optical sensor gets sharp images at up to 6,400 frames per second but the optimization of the speed of rotation is not covered in this work.

The procedure for counterfeit coin detection will be based on image processing algorithms applied to the common side of the two-Euro coin to avoid the huge diversity in the national side. Two optical sensors are used to get images of both sides of the coin under test. The optical sensors do not cover the complete image so they are placed in a relative radius form the center of rotation of the two-Euro coin. The optimal placement radius of the optical sensors will be analyzed lately in this work. The optical sensor can be used in counterfeit coin detection using two different alternatives: SQUAL based identification and template matching identification.

### A. SQUAL based identification

The SQUAL register gives information about the small changes (also called features) available for motion measurement in the current image of the optical sensor. This register is an indication of the roughness (or relieve) of the surface measured indirectly through the shadows enhanced by the lateral illumination applied. The SQUAL register has values from 0 to 169; a high value means that the image-processing algorithm used to detect motion will have more points to compare and the motion will be measured more accurately. The reading of the SQUAL register is compatible with the motion registers so sensor displacement and roughness can be read simultaneously.

**Figure 8.**
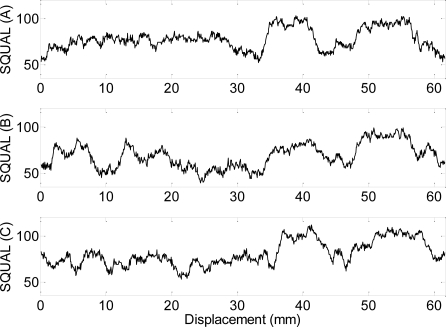
Dynamic evolutions of the values of the SQUAL register in a complete revolution of three different two-Euro coins.

Figure 8 presents the evolution of the SQUAL register in one rotation of the common side of three different coins (A, B and C) with the optical sensor placed at a radius of 10 mm from the center or rotation of a two-Euro coin. The sensitivity of the SQUAL value to the small imperfections originated in the coin by the daily use is extraordinary and every coin has its own footprint that is very repetitive and very different from coin to coin. The optical sensor has other internal registers as the SHUTTER and PIXELSUM but they are highly correlated with the values obtained in the SQUAL register. Therefore, the use of the values of the SQUAL register will allow individual coin recognition but are not valid for general counterfeit detection.

**Figure 9.**
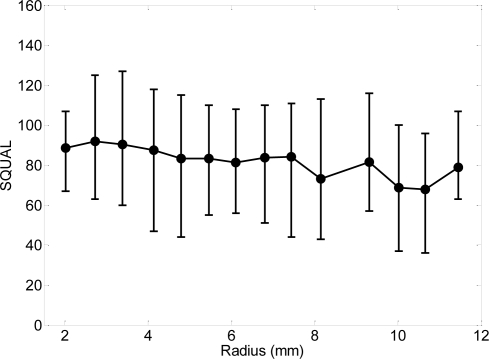
Average, maximum and minimum value of the SQUAL register obtained in one rotation of a two-Euro coin with the optical sensor placed at different radius.

Finally, Figure 9 shows the average, maximum and minimum value of the SQUAL register when the optical sensor is placed at different radius. Results shows very similar variations in all radius analyzed allowing a random placement over the coin.

### B. Template matching

The optical mouse sensor can be used as a real-time image acquisition device to obtain images from the common side of the two-Euro coin. The optical sensor only covers one part of the coin under test so the sequence of images acquired by the sensor must be angularly referenced for later analysis. This reference can be obtained using an additional encoder in the DC motor attached to the supplementary wheels (the method used in this work) or using the original motion detection functionality of the optical mouse sensor. This second option is only possible if one optical sensor is configured as a mouse (to measure angular motion [17]) while the other is used to get the sequence of images of the other side of the coin. Unfortunately, the mouse motion detector functionalities are stopped when reading the image acquired by the CMOS camera so this may require two rotations of the coin under test to get both coin sides but this feature can change in the near future. The real-time acquired images can be compared with a reduced set of images of a reference two-Euro coin using template matching algorithms for counterfeit detection.

**Figure 10.**
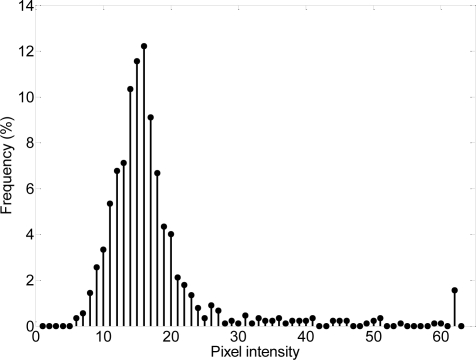
Typical histogram of an image of the common side of the two-Euro coin.

The first step in this detection procedure is to select a reduced set of reference images of a valid two-Euro coin for later template matching. There is no need to select a large set of images because most part of the coin has no relieve information resulting in a pure gray image. The procedure to select the reference images is based on a segmentation of the acquired images. Figure 10 shows the histogram of a typical image of the common side of a two-Euro coin. Pixel intensity values upper than 60 corresponds to white pixels in the image (intensity range is from 0 to 63) that can be considered relevant relieve information. Therefore, once a reference valid two-Euro coin is placed inside the detector, the two supporting wheels of the detector rotate the coin for complete real-time image scanning and relieve analysis.

**Figure 11.**
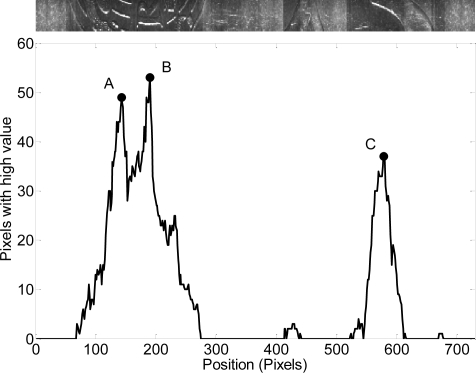
Number of white pixels found in the images of the common side of the two-Euro coin. The angular displacement was measured with a second optical sensor in the other side.

**Figure 12.**
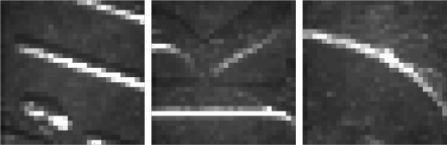
A (left), B (middle), and C (right) reference images selected as templates.

Figure 11 shows the relevant relieve information (white pixels) found in the acquired images during a complete rotation of a two-Euro reference coin; a composition of all image acquired is also shown in the upper part of the figure. The peaks of the data, points A, B, and C, depict the candidate reference images with relevant relieve information, show in Figure 12. During this measurement the optical sensor was placed at 7.5 mm from the center of rotation of the coin.

**Figure 13.**
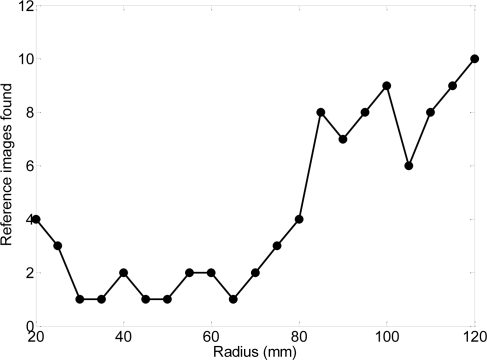
Number of reference images automatically selected depending on the placement radius of the optical sensor.

Figure 13 shows the number of reference images automatically selected for a two-Euro coin relative to the placement radius of the optical sensor. The number of reference images increases suddenly for radius upper than 8.5 mm because of the circular line defined by the union of the two metals of the coin. In general, any radius from 7.5 mm can be used for as a placement radius.

The selected images and their relative angular position are stored for later template matching in the memory of the microcontroller of the detector. The amount of memory needed to store the reference images is very low because the images have only 30 × 30 pixels with only one intensity layer enabling a true low cost implementation.

The procedure for vision-based counterfeit detection is as follows: the coin under test is rotated by the supporting wheels while taking images with the optical sensor. Each new image acquired is compared with all reference template images and the relative position of the best matching is stored for later use. The angular distance between the reference images is then compared with the angular distance defined by the locations of the best matching found. Finally, a threshold value is applied to the difference of these angular distances to make the classification of the coin.

## 5. System Validation

**Figure 14.**
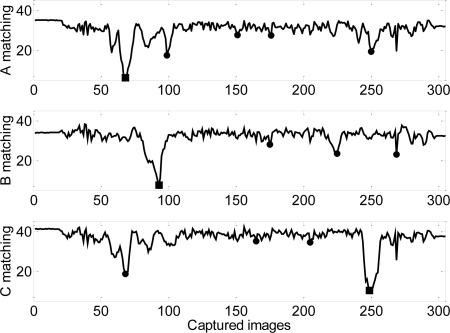
Evolution of the matching results in one rotation of three template images for a valid two-Euro coin. The solid squares depict the angular position of the best matching.

The final implementation of the counterfeit detector is based on the proposed template matching procedure. Figure 14 shows an example of the matching obtained for a valid two-Euro coin. The images acquired by the optical sensor are compared with three predefined template images (Figure 12) of the common side of a two-Euro coin minted after 2006. The absolute minima define the angular position of the best matching. The relative angular distance between the best matching of the first, second and third images are measured and compared with the relative angular distance between the original template images. The rule for valid classification requires individual template matching with a value lower than 10 (average intensity value) and a difference lower than 5° in the relative angular position between templates and images. Results shown in Figure 16 fulfill all rules for valid classification.

**Figure 15.**
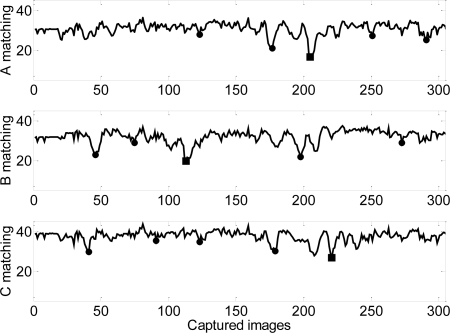
Evolution of the matching results in one rotation of three template images for an invalid two-Euro coin. The solid squares depict the angular position of the best matching.

Figure 15 shows an example of the matching obtained for a false two-Euro coin; in this case a legal coin from a country outside E.U. The real-time images acquired by the optical sensor are again compared with the three predefined reference images (Figure 12) of the common side of a two-Euro coin minted after 2006. Figure 15 shows that the best matching obtained for all templates have higher values than the predefined threshold and then the coin is automatically rejected.

Finally, the proposed counterfeit detection system will be validate with a representative number of coins corresponding to different sets of valid and invalid two-Euro coins. Table 1 shows the decision categories for valid and invalid coins. Automatic classification results will be validated with the classification results obtained with a trained and untrained human using only the vision sense (without touching or holding the coins).

Table 2 shows the comparative validation results obtained. The set of samples (and number) was valid coins (100), local classes (9), similar coins from other countries (36) and representative common classes of counterfeit coins (50) retired from circulation and offered for the test by the Departamento de Emisión y Caja of the Bank of Spain. The coins provided represent 15 of the most dangerous common classes labeled by the OLAF [21]. In general, results show that the proposed counterfeit detector has better performances than an untrained human but slightly lower than a trained. The results obtained for the untrained human depend largely on the development of the experimental procedure of the test. In this case we have awarded the volunteer that some coins can be false otherwise practically all of them would be accepted.

The proposed counterfeit detector has a 3% rejection rate of valid coins; this value corresponds to coins altered by abrasion that are usually retired from circulation when arriving to the banks. Local classes were 100% rejected because most of them are very bad copies. The coins from other countries with a similar shape, size and weight than the two-Euro coin were 100% rejected, increasing the rejection rate obtained in [10] for this specific case. Alternatively, most common classes provided by the Departamento de Emisión y Caja of the Bank of Spain for the test were accepted as valid coins as they are very good copies.

## 6. Conclusions

This work presents the implementation of a two-Euro counterfeit coin detector based on the image acquisition capabilities of the optical mouse sensor. The main advantages of this proposal are the inexpensive cost of the image acquisition system, the integration of the illumination system in the optical sensor, and the compact size design of the counterfeit detector. The main disadvantage is the need to rotate the coin to increase the area covered by the optical sensor but this operation can be accelerated using multiple chips in each side of the coin.

Two detection systems have been proposed and tested. The first procedure is based on the information provided by the internal SQUAL register (roughness information) that can be useful to obtain a unique foot-print of a particular coin but not for general counterfeit detection.

The second procedure is based on the image acquired by the optical sensor that can be used to implement a counterfeit detector based on template matching with reference partial images of a valid coin. Validation results show that valid coins are rejected if they are altered by abrasion (having less relieve); local classes (bad copies) and coins from other countries are 100% rejected as they are visually different; and common classes (good copies) are hardly identified as counterfeit but a trained human has similar acceptance ratio when identifying these coins using only the vision sense.

## Figures and Tables

**Table 1.** Decision categories for valid and invalid coins.

	**Acceptance**	**Rejection**
**Valid coin**	Correct detection	False rejection
**Invalid coin**	False acceptance	Correct rejection

**Table 2.** Validation comparative results.

		**Samples**	**Automatic Identification**		**Human Identification**			
					**Untrained Human**		**Trained Human**	
			Acceptance	Rejection	Acceptance	Rejection	Acceptance	Rejection
**Valid Coin**		100	97%	3%	84%	16%	100%	0%
**Invalid Coin**	Local classes	9	0%	100%	0%	100%	0%	100%
	Similar from other countries	36	0%	100%	81%	19%	0%	100%
	Common classes	50	76%	24%	92%	8%	74%	36%
